# Chitosan-Based Hydrogels for Bioelectronic Sensing: Recent Advances and Applications in Biomedicine and Food Safety

**DOI:** 10.3390/bios13010093

**Published:** 2023-01-06

**Authors:** Si Wu, Shijing Wu, Xinyue Zhang, Tao Feng, Long Wu

**Affiliations:** 1 College of Resources and Environmental Engineering, Wuhan University of Science and Technology, Wuhan 430081, China; 2 Hubei Key Laboratory for Efficient Utilization and Agglomeration of Metallurgic Mineral Resources, Wuhan University of Science and Technology, Wuhan 430081, China; 3 School of Food Science and Engineering, Key Laboratory of Tropical and Vegetables Quality and Safety for State Market Regulation, Hainan University, Haikou 570228, China

**Keywords:** chitosan, bioelectronic sensors, biomedicine, food safety

## Abstract

Due to the lack of efficient bioelectronic interfaces, the communication between biology and electronics has become a great challenge, especially in constructing bioelectronic sensing. As natural polysaccharide biomaterials, chitosan-based hydrogels exhibit the advantages of flexibility, biocompatibility, mechanical tunability, and stimuli sensitivity, and could serve as an excellent interface for bioelectronic sensors. Based on the fabrication approaches, interaction mechanisms, and bioelectronic communication modalities, this review divided chitosan-based hydrogels into four types, including electrode-based hydrogels, conductive materials conjugated hydrogels, ionically conductive hydrogels, and redox-based hydrogels. To introduce the enhanced performance of bioelectronic sensors, as a complementary alternative, the incorporation of nanoparticles and redox species in chitosan-based hydrogels was discussed. In addition, the multifunctional properties of chitosan-based composite hydrogels enable their applications in biomedicine (e.g., smart skin patches, wood healing, disease diagnosis) and food safety (e.g., electrochemical sensing, smart sensing, artificial bioelectronic tongue, fluorescence sensors, surface-enhanced Raman scattering). We believe that this review will shed light on the future development of chitosan-based biosensing hydrogels for micro-implantable devices and human–machine interactions, as well as potential applications in medicine, food, agriculture, and other fields.

## 1. Introduction

Flexible bioelectronic sensors have emerged to mimic biological functions, including human motion tracking [1,2], health monitoring [3,4], disease diagnosis [5], artificial bioelectronic tongues [6], and so on. Intrinsically, biology and electronics are essentially different, both in their mechanical and sensory properties. Electronic devices are solid and robust, while biological tissue is soft and elastic. On the other hand, their methods of signal transmitting are distinct from each other. Electromagnetic radiation makes long-distance communication possible, but this method does not exist in biological systems. Another challenge of bioelectronic sensors is to select reliable communication modalities at the interfaces connecting the device and biological tissue. Thus, it is of critical significance to bridge the gap between electronics and biology, with the end goal of fabricating a flexible bioelectronic interface and enlisting proper signaling modalities for bioelectronics.

Hydrogels have become competitive candidates due to their analogous tissue structure, excellent biocompatibility, and tunable functions [7,8,9,10]. The properties of hydrogels can be easily changed by external stimulus. For instance, chitosan forms hydrogels at a pH over 6.5; agarose and gelatin are dissolved at high temperatures; and alginate and calcium ions can form gels [11,12,13,14]. Therefore, hydrogels are able to act as an excellent interface for the perception and transition of biological signals.

Chitosan is a natural polymer derived from chitin, which exists widely in nature [15]. It is a prospective material for flexible bioelectronics, owing to its biosafety, chemical reactivity, similar water content, and Young’s moduli to tissue and cells, as well as its dynamic reconfigurable functions [16]. Biosensors with adhesive properties that can attach to wet and dynamic surfaces have drawn much attention in tissue engineering, human–machine interfaces, and wound dressings. As such, a carrier is required to connect biology and electronics. Chitosan exhibits antimicrobial and biocompatible properties, and it also serves as a good bio-adhesive [17]. Thus, chitosan-based hydrogels show great potential to construct a simple, biocompatible, and multifunctional interface between biology and electronics.

**Figure 1:**
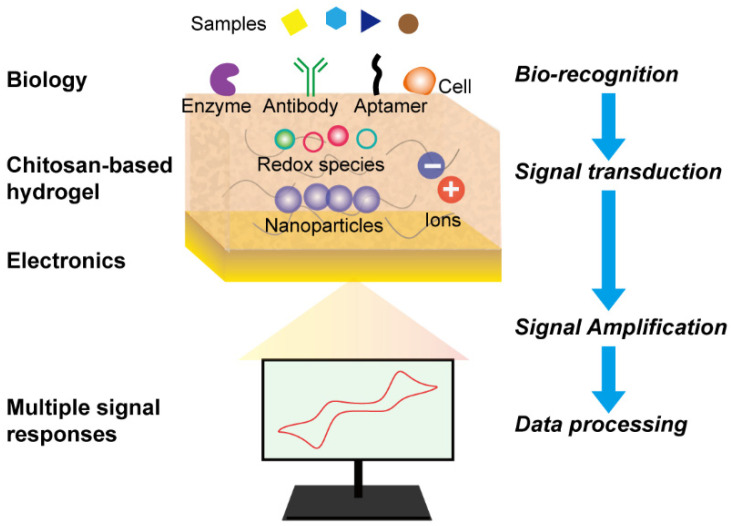
Schematic illustration of chitosan-based bioelectronic sensors.

Figure 1 demonstrates the mechanism of chitosan-based bioelectronic sensors from bio-recognition to data processing. Therein, chitosan-based hydrogel acts as a bridge to connect biology and electronics, and the analytes can be recognized by bio-elements such as antibodies and aptamers. Then, the interaction is transferred to optical/electronic signals, which are finally amplified into measurable outcomes. This review aims to summarize the fabrication approach and the interaction mechanism of chitosan-based composite hydrogels. We briefly classified the hydrogel bioelectronics into four types [18]: (i) electrode-based hydrogels, (ii) conductive materials conjugated hydrogels, (iii) ionically conductive hydrogels, (iv) redox-based hydrogels. Afterward, we discussed the recent progress of chitosan-based composite hydrogels in biomedicine and food applications. Finally, we concluded the challenges and perspectives of chitosan-based hydrogel bioelectronics in further applications.

## 2. Fabrication and Interaction Mechanism of Chitosan-Based Hydrogels

**Figure 2:**
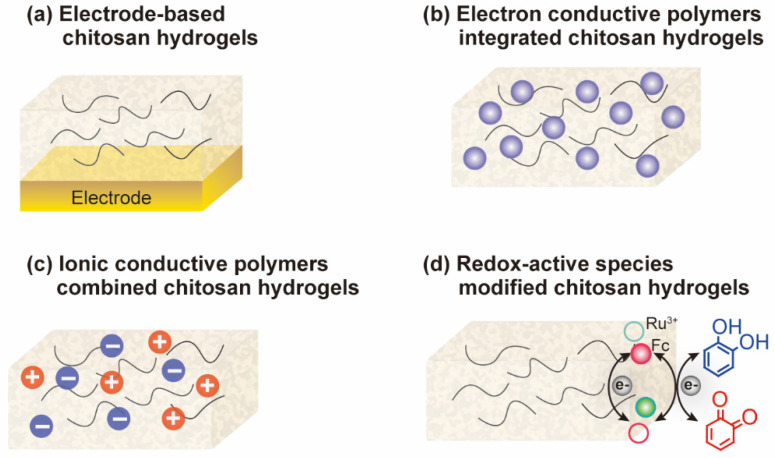
Chitosan-based hydrogels. (**a**) Electrode-based chitosan hydrogels. (**b**) Electron conductive polymers integrated chitosan hydrogels. (**c**) Ionic conductive polymers combined chitosan hydrogels. (**d**) Redox-active species modified chitosan hydrogels.

Chitosan is a kind of stimuli-sensitive polymer with reactive amine and hydroxyl groups, which endow chitosan-based hydrogels with functional properties (e.g., self-assemble, self-healing, shape memory, etc.) [19]. The interaction between hydrogels and biology strongly depends on the communication modality of bioelectronics. The conductivity of bioelectronic sensors is easily obtained by integration of metal nanomaterials [20,21,22,23], carbon-based nanomaterials [1,24,25,26,27], or other conductive polymers (e.g., polyaniline, polypyrrole) [28,29] into hydrogel networks. Furthermore, the redox-active polymers can also communicate with biological molecules through redox modality, which is widely used for signal processing in biology [30,31]. As illustrated in Figure 2, we summarized the hydrogel bioelectronics into four types: conductive electrode-based chitosan hydrogels, electron conductive polymers integrated chitosan hydrogels, ionic conductive polymers combined chitosan hydrogels, and redox-active species modified chitosan hydrogels.

### 2.1. Electrode-Based Hydrogels

**Figure 3:**
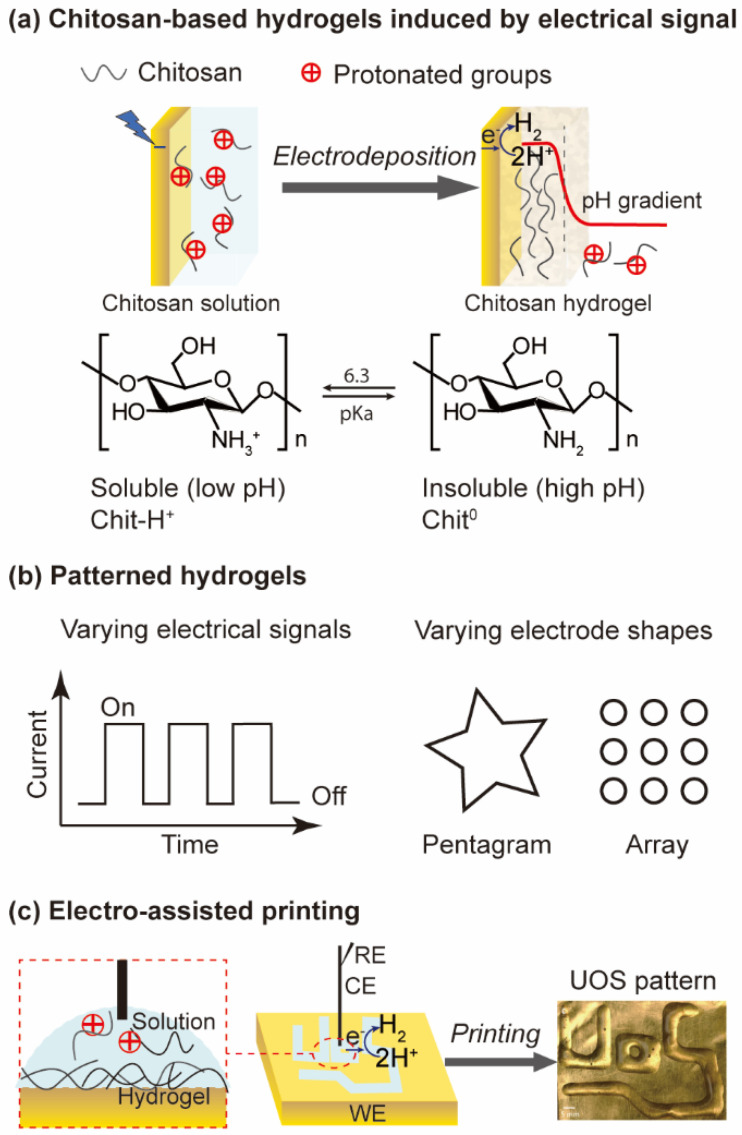
Electrofabrication of electrode-based chitosan hydrogels. (**a**) Chitosan-based hydrogels induced by electrical signals. (**b**) Patterned hydrogels formed by varying electrical signals or electrode shapes. (**c**) Electro-assisted printing of chitosan hydrogels. Adapted with permission from ref [33]. Copyright (2022) Springer Nature.

The pH cues generated by electrical signals can induce self-assembly of chitosan on the electrode surface to form hydrogels (Figure 3a). Thus, the hierarchical structure of chitosan-based hydrogels can be programmed and reconfigured by controlling electrodes and electrical inputs [32]. The electrochemical technique is simple, spatially controllable, and allows for bioelectronic applications. As shown in Figure 3b, patterned hydrogels are easily achieved by varying the electrode shapes and electrical signals, thus endowing the materials with unique functions and properties [33]. Electrodes can also be used to print patterns on hydrogels. As an example, Silva et al. introduced an electro-assisted printing approach to fabricate patterned chitosan and alginate conductive hydrogels on gold and ITO electrodes through covalent cross-linking and ionic polymerization mechanisms [34]. Figure 3c demonstrates that the UoS letters were printed using a high-molecular-weight chitosan at 1.8 V. The unique pH-responsiveness of chitosan allows it to form hydrogels easily on the electrode.

### 2.2. Conductive Materials Conjugated Hydrogels

The conductivity of hydrogels is easily achieved by integrating with conductive materials [35,36]. Metal nanoparticles and nanowires are intensively adopted to impart electrical conductivity to hydrogels. However, the heavy aggregation and poor stretchability limit their applications. Thus, the assembly of nanomaterials into the three-dimensional (3D) structure of hydrogels has attracted much attention. For instance, Yu et al. proposed a flexible and robust bioelectronic aerogel based on silver nanowires (AgNWs) assembled by a chitosan network and in situ synthesis of poly(acrylamide-sodium acrylate) hydrogel [37]. The composition of AgNWs enhanced the mechanical and antibacterial properties of the hydrogels, providing a new approach for the construction of 3D nanostructures into the hydrogels.

Another effective strategy for the fabrication of conductive bioelectronics is through in situ polymerization. For example, Duan et al. synthesized an ultra-stretchable, tough, and conductive chitosan-based hydrogel through in situ polymerization of acrylamide and aniline [29]. Lin et al. enlisted the graft copolymerization mechanism to fabricate highly conductive chitosan-polyacrylate/polyaniline hydrogels with good electrochemical performance [38]. The stretchability and conductivity properties of flexible hydrogels enable the bioelectronic applications. Some studies focused on the antibacterial properties of hydrogels. Based on the polymerization of pyrrole grafting, Zn^2+^ chelating, and borax cross-linking, Zhang et al. prepared conducting, self-healing, and antibacterial chitosan-based hydrogels with the ability to promote wound healing [39]. Conductive nanomaterials and polymers which conjugate chitosan hydrogels are promising for flexible bioelectronic interfaces due to their adhesive, mechanical, and conductive properties.

### 2.3. Ionically Conductive Hydrogels

Bioelectronic sensors based on electronic conductive hydrogels can communicate with biology using electrical signals. However, when the hydrogels are attached to skin or tissue, some metal nanomaterials may do harm to the cells. Moreover, conductive material-conjugated hydrogels are usually not transparent, and may limit their ability to visually detect the target products. Therefore, the strong demand for biocompatibility and the transparency of the hydrogels makes them essential in the bioelectronic applications on some special occasions (e.g., soft biosensors, optoelectronic sensors, etc.) [40]. The biology (e.g., the nervous and neuromuscular systems) can perceive signals through ionic electrical modality [31]. Thus, ionic conductive hydrogels can serve as an excellent transparent interface for the communication between biology and electronics.

Ionic hydrogels usually contain three important elements, which are water, polymer, and ionic conductors [41]. Ionic conductors are ionotronic, conductive, and water-dependent. Therefore, water is used as a medium to dissolve and transport ions, while polymers provide mechanical strength and 3D network structures of the hydrogels [42,43]. (Poly)electrolytes are usually introduced into hydrogels to acquire ion conductivity, including metal salt ions (lithium chloride, sodium chloride, potassium chloride, and aluminum chloride) [44,45,46,47,48,49,50] and Zwitterionic [51,52]. For instance, Khan et al. introduced a novel chitosan/poly(acrylamide-co-acrylic acid) hydrogel with self-healing and anti-freezing properties, while the ion conductivity was achieved through sodium chloride electrolytes. Thus, the employment of ionically conductive chitosan-based hydrogels has broadened the biomimetic applications, such as artificial bioelectronic tongues to detect food safety and quality, and smart actuators or robots to diagnose diseases in human body.

### 2.4. Redox-Based Hydrogels

There are three main communication modalities that biology uses for signal processing, including the ionic electrical modality, the molecularly specific modality and the redox modality [30,31]. Recently, redox modality has been verified as an effective approach for biology communication. For instance, reactive oxygen species (ROS) may cause oxidative stress, may promote the connections between the gut microbiota and the brain [53,54], and are relevant to some neurodegenerative diseases (Alzheimer’s and Parkinson’s disease), aging [55], etc.

**Figure 4:**
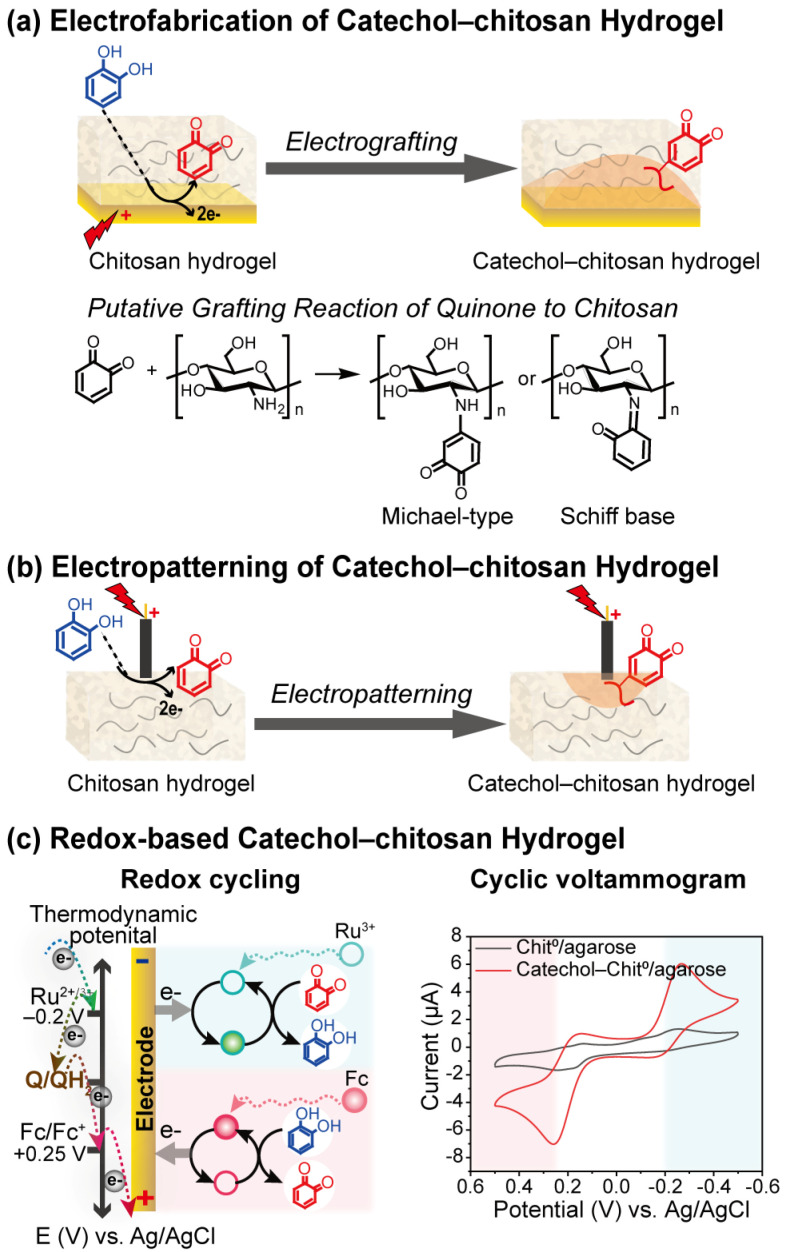
Redox-based hydrogels. (**a**) Electrofabrication of catechol–chitosan hydrogel. (**b**) Electropatterning of catechol–chitosan hydrogel. (**c**) The redox cycling and cyclic voltammogram of redox-based catechol–chitosan hydrogel. Adapted with permission from ref. [30]. Copyright (2019) ACS publications.

Phenolic compounds are abundant antioxidants in nature, and phenolic hydroxyl groups exhibit redox activity. As illustrated in Figure 4a, catechol–chitosan hydrogel can be fabricated on the electrodes by applying an oxidative potential. The phenolic hydroxyl group of catechol moieties can be easily oxidized under electrochemical conditions and grafted with the amino group of chitosan through a Michael type or Schiff base reaction [56]. Figure 4b demonstrates that catechol patterns can be printed on the surface of the chitosan-based hydrogel by moving the electrode and imposing positive voltage [57,58].

As shown in Figure 4c, catechol-modified chitosan hydrogel serves as a redox capacitor. The redox states of catechol are reversibly switchable in the presence of redox mediators. Thus, electrons can be transferred and stored between the electrode and the redox active catechol–chitosan hydrogel [59]. The catechol–chitosan hydrogel is able to interact with biological information, such as reactive oxygen species (NO, H_2_O_2_) [60,61], virulence factors of pathogenic bacteria (Pyocyanin) [62], bio-related reductants (NADPH [63], ascorbic acid [64], oxidants (O_2_) [65]), etc. For instance, Wu et al. fabricated a catechol–chitosan-based microfluidic channel modularity, which enables the in situ monitoring of information in biology [66]. Furthermore, the incorporation of nanoparticles enhances the performance of hydrogels and synergistically amplifies the redox-based electrochemical signals. For instance, Yan et al. added magnetic nanoparticles into a catechol–chitosan redox capacitor and allowed the chemical signals to be converted and amplified enzymatically and magnetically [67].

## 3. Functional Properties and Applications of Chitosan-Based Hydrogels

**Figure 5:**
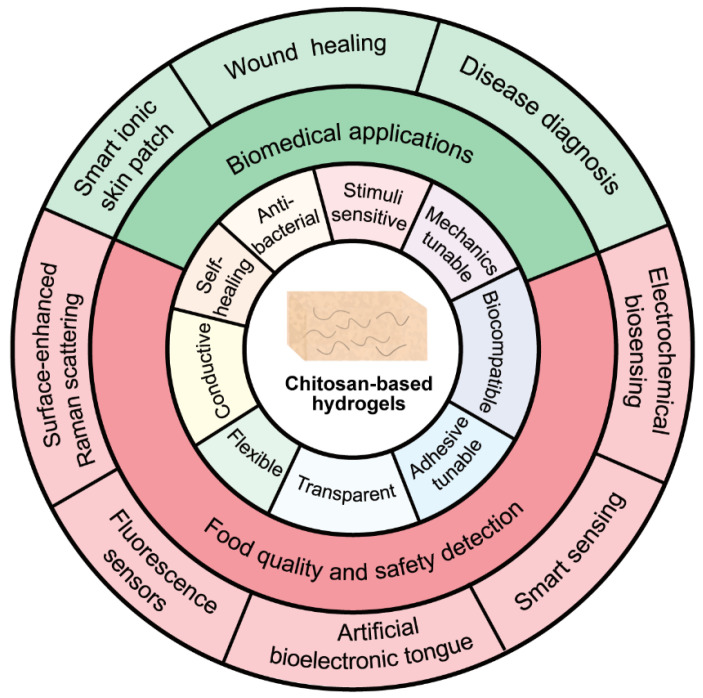
Properties and applications of chitosan-based hydrogels.

Biomimetic flexible, wearable, and implantable devices have attracted much attention in biomedical and food applications. Figure 5 gives a list of characteristics of chitosan-based bioelectronics, including stimuli-sensitive properties, tunable mechanics, good biocompatibility, strong tissue adhesion, optical transparency, flexible and conductive substrate, and self-healing and antibacterial functions [68].

These properties of hydrogels provide the capability to communicate between biology and electronics, which sheds light on its applications in biomedicine (e.g., smart skin patches, wood healing, disease diagnosis) and food safety (e.g., electrochemical sensing, smart sensing, artificial bioelectronic tongues to detect food quality and safety, fluorescence sensors, and the surface-enhanced Raman scattering approach for signal amplification).

## 4. Biomedical Applications

Chitosan-based hydrogels have been researched extensively in the bioengineering field for their simple fabrication, non-toxicity, and good biocompatibility [69]. The bioelectronic communication abilities of smart skin patch hydrogels enable human health diagnoses and signal interaction. The adhesive and antibacterial abilities of hydrogels promote wood healing and prevent secondary injuries. The biomedical applications of chitosan-based hydrogels as bioelectronic sensors are shown in Table 1.

### 4.1. Smart Ionic Skin Patch

Hydrogel exhibits similarities to human skin, and can be used as a smart ionic skin for health monitoring [79]. Moreover, the hydrogel bioelectronics offer the ability to sense external stimuli and transmit signals in biology. Much attention has been focused on hydrogel-integrated bioelectronics, including the performance of the hydrogels and the interaction between the hydrogel and the environment (e.g., external stimuli). However, it remains a challenge to prepare hydrogels that sense and respond to multiple signals simultaneously. Shi et al. developed a smart quaternized chitosan–polyacrylic acid hydrogel skin patch, which exhibited excellent conductivity, biosafety, and tunable adhesive ability [70]. The hydrogel skin patch offers bioelectronic temperature- and pressure-sensing functions that can even distinguish different physiological signals simultaneously. 

### 4.2. Wood Healing

The adhesive ability of biology and electronics together is also of critical importance for flexible bioelectronics. Existing tissue adhesives are cytotoxic, incompatible, and easily fall off [80]. Therefore, the fixation, biosafety and tunable adhesion of hydrogel are required on tissue or skin to avoid secondary damage and to promote wound healing [81]. The secondary damage and even inflammation may be caused when the hydrogel patch is removed from the skin [3,82]. Thus, the ability of hydrogels to reversibly adhere to wet and dynamic surfaces is critical in tissue engineering and wound healing. Mussel inspired biomimetic hydrogels have been extensively studied because of their reversible adhesive properties [83,84]. Catechol–chitosan-coated needles can even prevent blood loss in clinical experiments [72]. However, the adhesion generated from catechol chemistry is insufficient. Recently, bioinspired [44,51] and tough adhesives inspired by slugs’ defensive mucus [85] have been proposed to have strong and dynamic adhesive abilities. Tough hydrogel adhesion has been proposed based on topology connection and dissipation mechanics [86]. Li et al. established tough adhesives for various wet and dynamic surfaces through the synergy of two layers: chitosan, as the interfacial bridging layer, shows an adhesion energy of over 1000 J m^−2^; while the alginate–polyacrylamide hydrogel matrix layer can dissipate energy during deformation [85]. 

Considering the antibacterial and bioelectronic communication requirements for wound dressings, metal nanomaterials are incorporated into hydrogels. To avoid wound infection and inflammation, Pan et al. assembled AgNWs into chitosan networks and demonstrated a novel adhesive hydrogel conductor through in situ poly(acrylamide-sodium acrylate) hydrogel polymerization in a chitosan-AgNW aerogel structure. The aero-hydrogel hybrid conductor, with adhesive, biocompatible, and anti-bacterial capabilities, can be used as an excellent flexible bioelectronic interface for soft implantable devices [37]. 

### 4.3. Disease Diagnosis

The development of a bioelectronic sensor that can recognize the molecules in biological fluids and enable point-of-care detection is of critical importance. Catecholamines (e.g., dopamine, noradrenaline) are relevant to memory, cognition, and some diseases [87]. Shukla et al. fabricated a chitosan-based composite hydrogel embedded with carbon nanotubes, and explored two models for electrochemical information processing. The electrochemical sensor platform was able to quickly and efficiently detect the dopamine in urine samples without pre-treatment, which offers potential capability for clinical diagnosis and therapeutic monitoring of pheochromocytoma as well as other diseases [74]. However, the oxidation of dopamine may cause the contamination of the electrode, making it unable to be reused. Kim et al. proposed a catechol–chitosan–diatom hydrogel, which is flexible, self-healing, and conductive. The self-powered bioelectronic sensor can be used to diagnose body vibrations of Parkinson’s patients [75].

## 5. Food Quality and Safety Detection

In recent years, as the factories and industries have accelerated the economic boom, their wastes and byproducts have severe effects on the environment (e.g., atmosphere, water, oil), with various contaminants. The absorption of contaminants, especially heavy metals, by animals and plants is mainly driven by the level of contaminants in the environment and their accumulation and transformation in living organisms. Finally, some contaminants will be transferred to the human body through the food chain. On the other hand, with the common occurrence of food safety issues, food safety has become a highlighted public concern worldwide. Food contaminants such as pathogens, pesticides, and biotoxins induce food poisoning or diseases, which can cause serious threats to human health. Therefore, it is essential to develop rapid, sensitive, and accurate methods for the detection of such food contaminants. 

The traditional methods to monitor food quality and identify adulterated food products include HPLC, GC-MS, and chromatography [88,89]. Although these methods are effective, they are usually time-consuming, cost-prohibitive, and complex to operate. New devices (e.g., electrochemical sensors, cellphones) and methods (e.g., artificial bioelectronic sensing) are being developed and applied in food quality and safety detection [90,91].

### 5.1. Electrochemical Biosensing

The electrochemical biosensor platform has developed rapidly due to its simple operation, low cost, quick response, and high sensitivity. Chitosan hydrogel is able to immobilize biomolecules, which offers biosensing ability for the detection of nutrients (e.g., glucose, antioxidants) and toxic species (e.g., pesticides, mycotoxins, heavy metal ions) in food products [92]. Artigues et al. fabricated a glucose oxidase-immobilized and a titanium dioxide nanotube-coated chitosan biosensor, which showed great stability (20 days) and sensitivity (8.53 ± 2.39 µA mM^−1^) for glucose detection in various food samples [93]. Ochratoxin A (OTA) is a highly toxic mold metabolite that widely contaminates food products. Li et al. developed chitosan/dipeptide nanofibrous hydrogels modified with DNA and OTA aptamer. The concentration of OTA can be detected with a low detection limit of 0.03 ng mL^−1^. Moreover, the biosensor can be applied in white wine samples according to the impedance changes [94]. 

### 5.2. Smart Sensing

Smart phones are commonly used in our daily life. The high resolution of the camera and various apps in cellphones provides the ability to quickly analyze information. Enzyme-based biosensors can recognize the target markers and transduce the signal into an amplified and measurable signal. Wu et al. fabricated a catechol-patterned chitosan hydrogel that can identify high-fructose corn syrup in real samples. The grafted catechol moieties are able to accept electrons from glucose through an enzymatic reaction, and the changes in optical signal can be easily analyzed by smart phone imaging [95]. 

### 5.3. Artificial Bioelectronic Tongue

**Figure 6:**
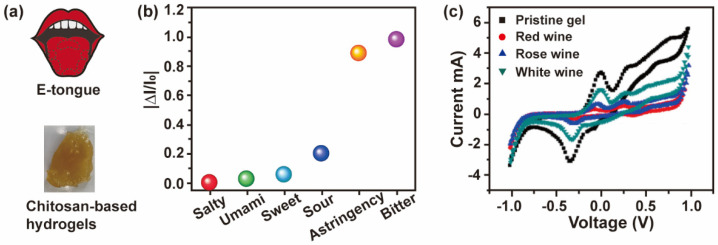
(**a**) Schematic and photograph of a chitosan-based bioelectronic sensor. (**b**) Selective sensing of five tastes and astringency by E-tongue. (**c**) Cyclic voltammetry measurement of real beverage samples. Adapted with permission from ref [6]. Copyright (2022) Elsevier.

The tongue is sensitive to five basic tastes, namely sweet, sour, bitter, salty, and umami. Various bioelectronic tongues are widely used to detect the quality and safety of foods [96,97]. Water-soluble flavors can be identified by changes in electrical signals generated by binding receptors or ion migration [98]. The concentration of different tastes is a key point in the food industry, as it affects the flavor of foods and beverages. Moreover, a bitter flavor is related to some poisons [99]. Therefore, the application of artificial bioelectronic tongues in food detection, the pharmaceutical industry, and environmental monitoring has attracted significant attention. As shown in Figure 6, Khan et al. fabricated a chitosan/poly(acrylamide-co-acrylic acid) hydrogel-based bioelectronic sensor to mimic the human tongue with a detection limit at the micromolar level. The bioelectronic tongue is flexible, self-healing, and anti-freezing, so it can also work in a −5 °C environment [6]. Nanostructured hydrogel sensors enable improved capabilities of the bioelectronic tongue. Salvo-Comino et al. proposed a bi-sensor using layer-by-layer construction of anionic sulfonated copper phthalocyanine, chitosan, and ionic liquid. Galactose oxidase was immobilized on the top of the layer, and offers the ability of detecting lactose concentration and freshness of milk [100]. 

### 5.4. Fluorescence Sensors

Due to their high sensitivity, strong selectivity, ease of use, good portability, and non-invasiveness, chitosan-based fluorescent probes have been widely used in the detection of food contaminants, especially of heavy metals. Different mechanisms have been reported on fluorescent probes interacting with food contaminants, including intramolecular charge transfer enhancement and fluorescence quenching. The former mechanism leads to a fluorescent “turn on” response, and the latter one gives “turn off” signals. 

Based on the Schiff base formation reaction, fluorescent chitosan hydrogels (FCHs) were obtained by introducing fluorophore BODIPY onto chitosan [101]. The combination of FCHs with Hg^2+^/Hg^+^ led to the quenching of the fluorescence, which showed strong selectivity for Hg^2+^/Hg^+^ with an adsorption capacity of 120.79 mg·g^−1^. With the functional modification of chitosan, they were multi-functionalized with strong selectivity, high adsorption capacity, hydrophily, etc. 

### 5.5. Surface-Enhanced Raman Scattering

SERS, short for surface-enhanced Raman scattering or surface-enhanced Raman spectroscopy, is a surface sensitive technique coupled with a rough metal surface, known as a SERS substrate. The incident laser stimulates the electrons on the SERS substrate surface to produce surface plasmon resonance with surface-attached molecules, which can greatly enhance the Raman signal. As a rapid, non-destructive, and sensitive method, SERS has been widely applied in the detection of food contaminants.

A biosynthesis method was proposed for the preparation of Au-embedded chitosan (Au@CS) by adding AuCl_4_ ions into chitosan [102]. Using this method, the flexible Au@CS acted as a reliable and sensitive SERS substrate, which enabled label-free detection of melamine in milk and thiamethoxam in fruit peels, with limits of quantification of 1.5 mg/kg and 0.001 mg/kg, respectively. Furthermore, a labeled SERS aptasensor was proposed for the detection of *E. coli* based on silver nanoparticles and *E. coli* aptamer (Apt)-modified chitosan (Apt-Ag@CS) [103]. When Apt-Ag@CS was further incubated with *E. coli,* 4-mercaptobenzoic acid (4-MBA), and apt-modified gold nanostars, it allowed for specific Raman signal amplification and detection of *E. coli*, with a detection limit of 3.46 CFU/mL. Chitosan-based SERS substrates provided a new direction for the detection of *E. coli* in practical food samples.

## 6. Conclusions and Future Prospects

Chitosan hydrogels serve as an excellent interfacial material to bridge biology and electronics, and have the capability for bioelectronic communication due to their biocompatibility, stimulus response, and tissue-like structure. Compared to other materials, chitosan-based hydrogels are simple to fabricate (physical and chemical methods), reconfigurable (reversible structure), and easy to modify (functional groups), so they have unique properties and behaviors (e.g., self-healing, adhesion, conductivity, etc.). This review illustrates the fabrication and mechanism of four types of chitosan-based hydrogel bioelectronics, including electrode-based hydrogels, conductive materials conjugated hydrogels, ionically conductive hydrogels, and redox-based hydrogels. The combination of different materials imparts functional properties to the hydrogels. For instance, the integration of nanoparticles enhances the mechanical properties and conductivity of the hydrogel, the modification of catechol provides redox activity and adhesion of hydrogel, etc. The functional properties of chitosan-based composite hydrogels give it its potential application in biomedicine and food safety. Chitosan-based hydrogels can be used as smart skin patches, wound dressings, or disease diagnosis biosensors. In food safety inspection and quality supervision, chitosan-based hydrogels can be used as a smart sensor to verify adulterated or fake foods, and as artificial bioelectronic tongues to detect food quality and concentration. 

However, there are still some limitations for the broad application of chitosan-based hydrogels. The primary one is the impact of the addition of nanoparticles on biosafety. Therefore, the concentration and cytocompatibility of different nanoparticles should be investigated. The network structure of the hydrogel is also beneficial for the encapsulation and immobilization of nanoparticles. The second is that the reusability of biosensors needs to be improved. This requires elucidating the contamination mechanism of electrodes and constructing sensor interfaces with anti-fouling capability. Additionally, the ability of chitosan-based bioelectronic sensors to access biological information and actuate signals requires further investigation. A mediated electrochemical probing system and information analysis offers an opportunity for biodevice communication [58].

In the future, chitosan-based hydrogels are promising as self-powered biosensors that can harvest energy from living organisms, and can also be used for health monitoring and real-time monitoring [104]. Its potential applications include microchip sensors for wireless communication and wireless electricity charging [5], as well as soft implantable biosensors that perform multiple tasks. Moreover, human–machine interaction is expected to be achieved through bioelectronic communication [105]. It is expected that multi-scale networked and automated monitoring will be achieved through machine learning by connecting materials, device design, and artificial intelligence. We believe that chitosan-based composite hydrogels will overcome these challenges and enable widespread applications in biomedical, food, agricultural and other fields in the future.

## Figures and Tables

**Table 1:** Chitosan-based bioelectronic sensing and its biomedical applications.

Materials	Hydrogel Types	Interaction Mechanisms	Properties	Applications	Ref.
Quaternized chitosan/polyacrylic acid hydrogel	Conductive polymers combined hydrogel	Polymerization, physical cross-linking	Mechanic tunable, adhesion reversible, pH sensitive, thermosensitive, biosafe, self-healing, conductive	Smart ionic skin patch	[70]
Chitosan/collagen-graphene oxide quantum dots hydrogel	Combined biopolymeric conductive hydrogel	Blending, condensation reaction	Biocompatible, injectable, thermally stable, promotes gene expression	Cardiac healing	[71]
Catechol–chitosan hydrogel	Redox-active hydrogel	Chemical cross-linking	Adhesive, self-sealing, hemostatic	Hemostatic needle coating	[72]
Chitosan-polypyrrole/Zn-functionalized chitosan/poly(vinyl alcohol) hydrogel	Conductive polymers combined hydrogel	Polymerization, chemical and physical cross-linking	Stretchable, flexible, self-healing, biocompatible, antibacterial	Chronic Wound Treatment	[39]
Polydopamine- carboxymethyl chitosan hydrogel	Gold electrode-based hydrogel	Polymerization, chemical cross-linking	Biocompatible, non-immunogenic, flexible, conductive, antioxidant, adhesive	Real-time wound monitoring	[73]
Hyperbranched Polyglycidyl ether /chitosan/ human-like collagen/MXene sheets/graphene hydrogel	Conductive polymers combined hydrogel	Polymerization, chemical and physical cross-linking	Flexible, antibacterial, electroactive, bio-adhesive, self-healing, hemostatic	Wound treatment, health monitoring	[74]
Quaternized chitosan/oxidized dextran/tobramycin/polydopamine@polypyrrole hydrogel	Conductive polymers combined hydrogel	Polymerization, chemical and physical cross-linking	Transparent, antioxidant, antibacterial, conductive, self-healing	Drug Release, wound healing	[75]
Chitosan quaternary ammonium salt/ sodium alginate hydrogel	Polyelectrolyte composite hydrogel	Physical cross-linking	Flexible, conductive, biocompatible, adhesive, hemostatic	Wound healing	[76]
Chitosan/carbon nanotubes hydrogel	Gold electrode-based hydrogel	Physical cross-linking	Conductive, redox active	Point-of-care testing for tumors	[77]
Catechol–chitosan-diatom hydrogel	Ionically conductive hydrogel	Chemical and physical cross-linking	Stretchable, skin-attachable, biocompatible, self-healing, self-powered	Real time health monitoring	[78]

## Data Availability

No new data were created or analyzed in this study. Data sharing is not applicable to this article.
